# Cyanobacterial extract mediated biogenic synthesis of NiO/CoO nanocomposites for enhanced photodegradation of crystal violet dye

**DOI:** 10.1038/s41598-026-66973-5

**Published:** 2026-08-20

**Authors:** Mohamed H. H. Ali, Mohamad S. Abdelkarim, Soaad A. Sabae

**Affiliations:** 1 Chemistry Lab, National Institute of Oceanography and Fisheries (NIOF), Cairo, Egypt; 2 Hydrobiology Lab, National Institute of Oceanography and Fisheries (NIOF), Cairo, Egypt

**Keywords:** Biogenic synthesis, NiO/CoO nanocomposites, Cyanobacterial extract, Photodegradation, Crystal Violet, Chemistry, Environmental sciences, Materials science

## Abstract

The continuous discharge of synthetic dyes into the aquatic environment poses a serious ecological threat, particularly biodegradable toxic cationic dyes such as crystal violet (CV). Consequently, developing effective and sustainable wastewater treatment methods is essential. To address the current research gap in green synthesis of multi-metal oxides heterostructures, this study provides a novel green synthesis route for nickel oxide/cobalt oxide (NiO/CoO@CM) nanocomposites using cyanobacterial extract, where the bioactive extracts act as a natural reducing and stabilizing agent for highly efficient synthesis. Both chemically synthesized NiO/CoO and biogenically NiO/CoO@CM nanocomposites were characterized using Brunauer-Emmet-Teller (BET), ultraviolet-visible diffuse reflectance (UV-Vis DRS), scanning electron microscopy coupled with energy dispersive X-ray spectroscopy (SEM-EDX), transmission electron microscopy (TEM), X-ray diffraction (XRD) and Fourier transformation infrared spectroscopy (FTIR). Characterization findings revealed that the biogenic NiO/CoO@CM showed higher surface area (38.97 m^2^/g), smaller crystallite size (27.05 nm), a narrowed band gap energy (2.9 eV) and abundant biofunctional groups. Under the optimized conditions (pH = 9, 90 min contact time, 0.1 g/50 mL catalyst dosage), the biogenic NiO/CoO@CM catalyst achieved 96.5% crystal violet (CV) degradation, significantly outperforming the 79.2% removals efficiency observed for chemically synthesized NiO/CoO. Isotherm and kinetic modeling reveled that Freundlich isotherm and pseudo-second order kinetics models best described the degradation process, indicating heterogeneous surface adsorption dominated by chemisorption. These findings confirm the novelty and potential of cyanobacterial extract-mediated synthesis as a cost-effective, eco-friendly and highly efficient technique for sustainable remediation of dye contaminated wastewater.

## Introduction

Synthetic dyes play an essential role and widely used in the different industrial purposes, such as plastics, paper, cosmetics and textiles, because of their vibrant colors, high stability and cost effectiveness^1,2^. Among these, crystal violet (CV) is a synthetic cationic triphenylmethane dye widely utilized in textile dyeing, medical antiseptic and biological staining. The discharged of untreated CV in wastewater poses severe threats to aquatic ecosystems and human health^3^. The international agency for Research on Cancer (IARC) classifies CV as a group 2B possible human carcinogen, whereas its major metabolites, Leucogentian violet (LCV) showed pronounced mutagenic and potential genotoxic effects. Long term exposure to LCV may induce genotoxic damage, through oxidation of DNA, chromosomal deviations, and hepatic tumorigenesis^4^. a These compounds highly bio-accumulates across various trophic levels, exerting chronic ecotoxicity and disruption of endocrine glands in aquatic organisms^5^. Thus, the regulatory frameworks of European Union have adopted a strict zero tolerance limits for CV and LCV residues in aquaculture products, some cosmetics and veterinary applications^6^. Given these environmental and public health concerns, the development of efficient and effective remediation technique to mitigate these adverse impacts has become an urgent necessity.

Conventional physical, chemical and biological wastewater treatment approaches often exhibit some limitations in the complete degradation and efficient removal of organic compounds. Physical treatment techniques produce certain secondary pollutants, e.g. sludge^7^. Chemical treatments frequently involve high energy consumption, low efficiency and release some non-renewable by-products, whereas biological treatments require long treatment times and low efficiency^8,9^. Therefore, there is an urgent need to develop new, promising, cost effective and sustainable technologies to treat the contaminated wastewaters, particularly with synthetic dyes^10^. Among them, photocatalysis methods using nanoparticles (NPs), especially green synthesis applying plant, algal, and fungal extracts, present viable, effective, and sustainable replacements for the specific elimination of such pollutants^11,12^.

Among the various photocatalytic metal oxide nanoparticles, nickel oxide (NiO) and cobalt monoxide (CoO) have possess considerable interest because of their exceptional properties, notably their remarkable stability and efficient capacity for generating reactive oxygen species (ROS) under light irradiation^13^. The bulk NiO is an antiferromagnetic p-type semiconductor (bandgap 3.1–4.0 eV), the nanoscale NiO exhibit superparamagnetism at room temperature due to uncompensated its surface spins. Its widespread application in electronic and biomedical fields due to its magnetic properties, catalytic efficiency and antibacterial characteristics^14,15^. Additionally, NiO is used in various industrial applications, including energy storage, sensors, solar cells, and memory storage devices^16^. Conversely, cobalt monoxide (CoO) is frequently utilized in gas sensors and the production of lithium batteries because of its low energy band gap **(**2.2–2.8 eV), pronounced strong redox activities, and chemically stable structure^17^. CoO nanoparticles could found in two crystalline phases: the thermodynamically stable cubic rock salt and hexagonal wurtzite structures^18^.

The integration of NiO and CoO into a stable mesoporous NiO/CoO heterojunction nanocomposite is considered a highly promising photocatalyst for the remediation of the organic pollutants, owing to its unique electronic structure and efficient charge transfer capabilities. The formation of a p-p heterojunction at the NiO-CoO interface enhances the effective separation of photogenerated electron hole pairs leading to suppressing their rapid recombination^19^. The NiO/CoO exhibits a relatively low bandgap of 2.02 eV with coexistence of Ni²⁺/Ni³⁺ and Co²⁺/Co³⁺ redox couples, associated with abundant oxygen vacancies, provides additional active sites that enhancing the degradation efficiency^20^. The synergistic interaction between NiO and CoO enables rapid charge transfer and prolongs the charge carrier lifetimes leading to enhanced generation of reactive oxygen species (•OH and •O_2_^−^)^21^.

The photocatalytic efficacy is further improved through green synthesis of nanoparticles using plant or algal extracts, offering a more ecologically friendly and sustainable approach^22–24^. Bioactive compounds present in cyanobacterial extracts including proteins, pigments, flavonoids, and polysaccharides, act as both reducing and stabilizing agents^25^. These bioactive compounds introduce new functional groups onto the catalyst surface, which promote dye degradation and enhance their photocatalytic performance^26^.

Algae represent highly efficient agents for the production of metallic nanoparticles, due to their exceptional capacity to accumulate metals and decrease metal ions, which has earned them the designation “bio-nano factories”. The use of both live and dried biomass highlights the adaptability of microalgae, which are essential to global biodiversity. Species such as *Lyngbya majuscule* and *Chlorella vulgaris* used to synthesis of nanoparticles economically and readily scalable for large-scale production. Algae as *Spirulina platensis and Spyrogira insignis* are comparatively easy to handle, less toxic, and less detrimental to the environment; their synthesis can be done in simple aqueous solutions under normal pH, temperature and pressure. This has led the emergences of phyco-nanotechnology as a distinct research field^27^. Cyanobacterial mats inhabiting in extreme desert habitats exposure to intense light radiation, thermal fluctuation, hypersaline waters, and limited nutrient^28^. Such harsh conditions stimulate the produce of a wide range of bioactive metabolites, including polysaccharides, phenolic compounds, pigments, peptides and antioxidants which enhance their resilience compared to other strains growing in ordinary environmental conditions. These metabolic richness makes extremophilic cyanobacteria as promising bioresources for environmental remediation and nanotechnological applications^29^.

Several recent studies have demonstrated the significant efficacy of green-synthesized CoO and NiO in the photodegradation of various organic dyes. For instance, Sarvalkar et al.^30^ synthesized Co_3_O_4_ using *Aloe barbadensis* extract, achieving 97.3% crystal violet (CV) dye elimination from aqueous solution. Similarly, rGO/ZnCo_2_O_4_ exhibited 99% degradation efficiency for CV dye^11^. Samuel et al.^31^ reported a 98% removal efficiency of acid blue-74 dye using green CoO synthesized with grape Jumbo Muscadine (*Vitis rotundifolia*). Shanmuganathan et al.^32^ utilized *Curcuma longa* root extract to synthesize CoO for removal of methyl orange, methyl red and methyl blue, achieving a maximum removal efficiency of approximately 60%. Piriyadharsini et al.^33^ reported removal efficiencies of approximately 94% and 96% for methylene violet and methyl orange, respectively using CoO synthesized with *Citrus Medica* L. extract. Additionally, Rajkumar and Navamathavan^20^ reported 85.5% degradation efficiency for methylene blue dye using a NiO/CdO/Co_3_O_4_ nanocomposite. Ikram et al.^34^ achieved 82.2% degradation of crystal violet using NiCo_2_O_4_ nanoflakes, while the degradation efficiency of methylene blue dye reached 98.1% within 90 min using biogenic CuO-NiO fabricated with *Macroptilium lathyroides* leaf extract^35^. Under the illumination of a High-Pressure Mercury Vapor (HPMV) Lamp, NiO nanoparticles acting as a catalyst eliminated 76.92% of methylene blue dye in 50 min and 75.41% of methyl orange dye in 25 min. These findings indicate that, when employing eco-friendly NiO nanoparticles under HPMV lamp light can effectively transforms MB and MO dyes into harmless byproducts^36^.

While numerous studies have investigated the photocatalytic efficiency of individual metal oxides; the present manuscript focuses on the synergistic integration of two metal oxides, using cyanobacterial extract as a sustainable and eco-friendly alternative to conventional chemical routs or fabrication of single-metal oxide. Although the effects of single metal oxide have been extensively investigated, the incorporation of multiple metal oxides with algal or bacterial extracts remains a largely unexplored. To address this gap, the current work uses cyanobacterial extract as reducing, capping, and stabilizing agents to synthesize highly efficient photocatalytic nanoparticles with enhanced organic pollutant degradation capabilities. Additionally, the proposed technology contributes to the advancement of sustainable wastewater treatment applications by offering a low-energy, economical, and sustainable technique.

## Materials and methods

### Chemical and reagents

Cobalt chloride hexahydrate (CoCl_2_⋅6H_2_O), nickel nitrate hexahydrate (Ni(NO_3_)_2_.6H_2_O), crystal violet (C_25_H_30_ClN_3_), and sodium hydroxide (NaOH) were used for preparation of Nio/CoO nanocomposites. All chemicals were of ultra-pure quality with a purity grade of ≈ 99% and were sourced from Merck, used without any additional purification. Cyanobacterial mats were collected from a hypersaline lagoon in Siwa Oasis, located in Western Desert of Egypt. The mats were carefully rinsed with distilled water to eliminate any leftover sediment and debris. Subsequently, they were dried in an oven (Memmert GmbH) at 45 °C overnight. Once dried, the biomass was crushed and passed through a 63-mesh sieve, with the fine particles kept in clean bags for later use.

### Cyanobacterial mats extract

The extremophilic cyanobacterial mats utilized in this study were collected from the Siwa Oasis, located in the Western Desert of Egypt, where they thrive in a hypersaline lagoon (Salinity > 130‰). To extract the bioactive compounds, 10 g of finely ground mat biomass was combined with 50 mL of distilled water. The resulting mixture was subjected to sonication at 45 °C for 60 min to facilitate the extraction of the intracellular components. Following sonication, the mixture was heated to 80 °C while being continuously stirred magnetically at 500 rpm for 6 h. Subsequently, the suspension was centrifuged at 10,000 rpm for 15 min to separate the solid residues. The supernatant, containing the active constituents, was carefully collected and stored at -4 °C for further use.

### Chemical and green NiO/CoO photocatalyst preparation

The synthesis of NiO/CoO nanocomposite was carried out according the procedure established by Zhou et al.^37^ through a chemical co-precipitation method. 100 mL of 0.1 M cobalt chloride hexahydrate (CoCl_2_⋅6H_2_O) was carefully added dropwise to 100 mL of 0.1 M nickel nitrate hexahydrate (Ni(NO_3_)_2_.6H_2_O) under continuous magnetic stirring at 500 rpm and 80 °C. To induce the co-precipitation of metal hydroxide, the pH of the mixture was gradually raised to 10–11 by dropwise addition of sodium hydroxide solution. The reaction mixture was then continuously stirred for six to eight hours at 80^o^ C. The obtained precipitate was thoroughly washed repeatedly to eliminate impurities, separated by centrifugation at 10,000 rpm for 15 min, and subsequently calcined at 600 °C in a furnace to obtain the NiO/CoO nanocomposite.

**Fig. 1:**
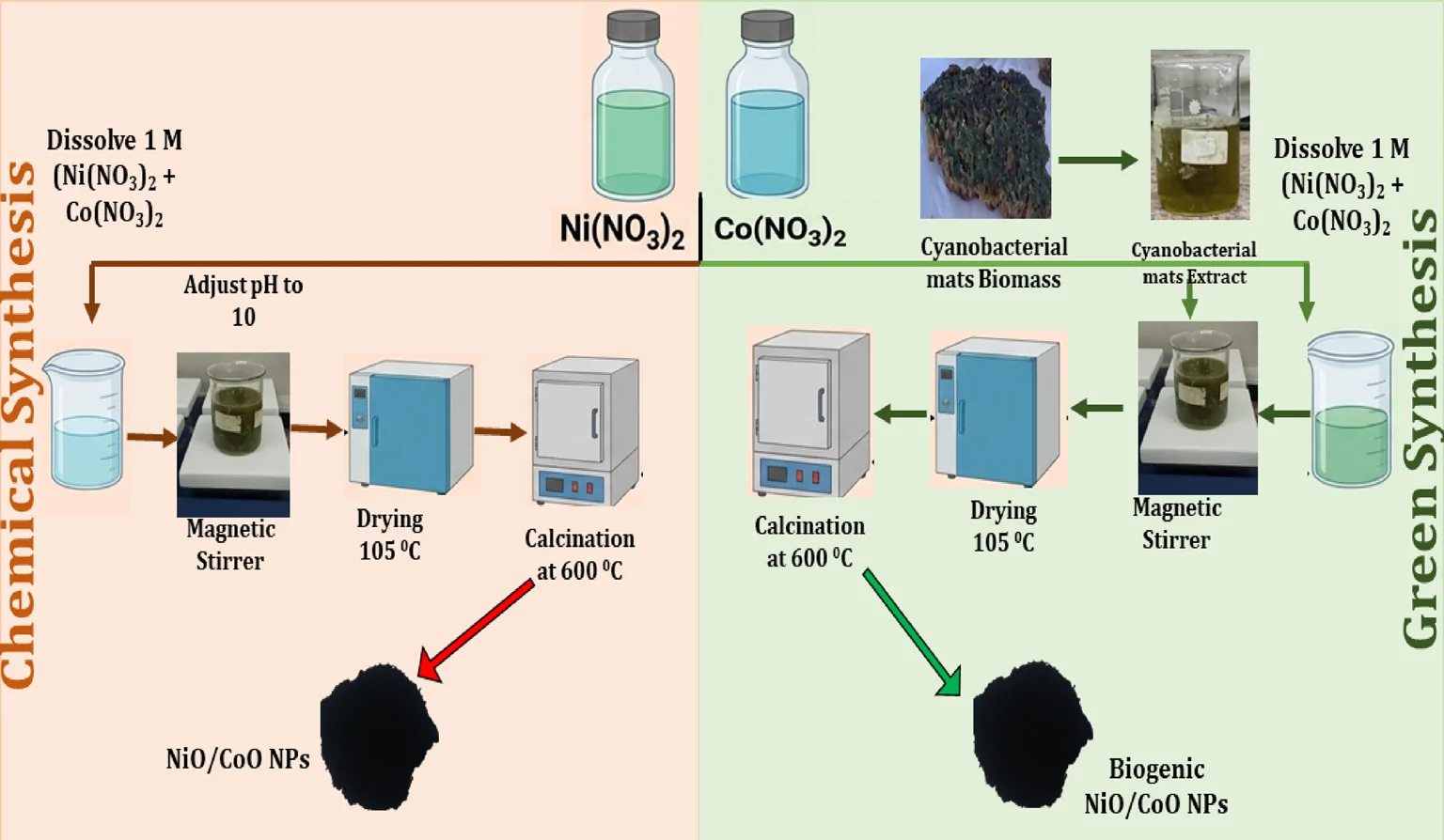
Schematic illustration for the preparation routs of chemically and biogenically NiO/CoO nanocomposites.

For the green synthesis of NiO/CoO@CM nanocomposite, cyanobacterial mats extract - rich in bioactive compounds such as polysaccharides, phenolic compounds, and proteins- was employed as a reusing and capping agent. Here, 50 mL of the extract was added slowly to a mixed solution of 0.1 M CoCl_2_⋅6H_2_O and 0.1 M Ni(NO_3_)_2_.6H_2_O under continuous magnetic stirring at 70 °C for 6–8 h. The resulting precipitate washed to remove any impurities, centrifuged at 10,000 rpm for 15 min, and calcined at 600 °C to yield the NiO/CoO@CM nanocomposite (Fig. 1).

### Characterization

The photocatalysts were thoroughly characterized in order to assess their optical, structural, and morphological characteristics. Nitrogen adsorption-desorption isotherm were recorded at 77 K using Belsorp MINI X surface area analyzer to determined the pore size distribution and specific surface area via the Brunauer–Emmett–Teller (BET) technique. Using a Shimadzu UV-2600 spectrophotometer equipped with an integrating sphere, diffuse reflectance spectroscopy (DRS) was used to determine the band gap energy (E_g_) and examine the optical characteristics. The optical band gap energy (E_g_) was determined from the UV-Cid diffuse reflectance spectra using The Kubelka-Munk function and the Tauc relation assuming a direct allowed electronic transition. (Eq. 1)

1 $$ ({\mathrm{F}}\left( {\mathrm{R}} \right){\mathrm{hu}})^{{\mathrm{2}}} = {\text{ A}}({\mathrm{hu}} - {\text{ E}}_{{\mathrm{g}}} ) $$

A is a constant, hυ represent the photon energy, h is Planck’s constant, F(R) is the Kubelka-Musk function, and E_g_ is energy gap.

The surface morphology and particle aggregation of the photocatalyst were examined using a FEI Quanta FEG 250 scanning electron microscopy (SEM). Coupled with the SEM, energy-dispersive X-ray spectroscopy (EDX) was employed to determine the elemental composition and confirm the presence of Ni, Co, and O within the samples. The particle size, and shape were determined using Tecnai G2 Supper Twin, High-resolution transmission electron microscopy. XPS analysis was conducted on an ESCALAB 250Xi spectrometer (Thermo Fisher Scientific, Waltham, MA, USA) using monochromated Al Kα radiation (hν = 1486.6 eV). X-ray photoelectron spectroscopy (XPS) analysis was conducted using Thermo Scientific K Alpha spectrometer (Thermo Fisher Scientific, Waltham, MA, USA) equipped with monochromated Al Kα radiation (hυ = 1486.6 eV). Structural properties were investigated via X-ray diffraction analysis on a D2 phaser 2nd generation Bruker diffractometer utilizing Cu-Kα radiation (λ = 1.5406 Å, 40 kV). Diffraction patterns were recorded over 2θ ranging from 20⁰ to 80⁰ at scan speed of 10⁰ /minute. Crystalline phases were identified by distinct diffraction peaks corresponding to NiO and CoO. The average crystallite size was determined using the Scherrer equation (Eq. 2).

2 $$\:D=\frac{K{\uplambda\:}}{{\upbeta\:}\mathrm{C}\mathrm{o}\mathrm{s}{\uptheta\:}}$$

where D represent mean crystallite size, K is the dimensionless shape factor (= 0.9), λ denote the X-ray wavelength (1.5406Å), θ is the Bragg diffraction angle, and β signifies the full width at half maximum (FWHM).

Finally, an ATR-FTIR spectrometer (Thermo Niclot, 50) was used to identify the metal-oxide bonds in the nanocomposites and the functional groups produced from the cyanobacterial extract. A spectral range of 400 to 4000 cm⁻¹ was used for the investigation.

### Degradation of crystal violet (CV)

The degradation of crystal violet (CV) dye was systematically designed to assess the catalytic efficiency of NiO/CoO and NiO/CoO@CM nanocomposites under certain specific factors including solution pH, contact time, catalyst dosage and initial dye concentration. All experiments were conducted at room temperature under UV light (λ = 365 nm) in a reactor fitted with six 45 W mercury lamps equipped with a cutoff filter providing irradiation ≥ 365 nm, with constant stirring at 300 rpm.

Solution pH (4–10) was examined by adjusting the solution with 0.1 M of NaOH or HCl, using fixed CV dye concentration (100 mg/L, pH 7) and a catalyst dose (100 mg per 50 mL) and irradiating time (90 min). Irradiation time was investigating over regular interval of 15–150 min with a 50 mL dye solution (100 mg/L, pH 7) and a 100 mg dose. The Catalyst dose was varied (20 to 120 mg per 50 ml) at pH of 7 for 90 min, while the initial CV concentration was tested across a range of 10–120 mg/L. Before UV exposure, Prior to illumination, all samples were stirred in the dark for thirty minutes to achieve the adsorption-desorption equilibrium. Post irradiation period, 10 mL samples were collected and centrifuged at 12,000 rpm for 8 min to separate catalyst particles, the remaining concentration of the dye was then measured spectrophotometrically at λ = 590 nm using a Jenway 6800 UV / VIS spectrometer). To ensure the statistical reliability and minimize errors, all photocatalytic degradation experiments were repeated three times under the same conditions, the reported data representing the average of these replicates. The photocatalytic capacity (q_ₑ_) and the overall degradation efficiency (R%) were determined using Eqs. 3 & 4.

3 $$\:{q}_{e}=\:\frac{{(C}_{0}-{C}_{t})\:x\:V}{M}$$

4 $$\:R\%=\:\frac{{C}_{0}-{C}_{t\:}}{{C}_{0}}\:x\:100$$

where q_e_ represent the degraded dye concentration (mg/g), R% represent the percentage of dye removal, V refer to solution volume (L); C_0_ and C_t_ signify the concentration of CV dye at the initial and equilibrium; and M is the mass of the catalyst (g).

The mineralization of crystal violet dye was evaluated using both NiO/CoO and biogenic NiO/CoO@CM nanocomposites under optimized photodegradation conditions (50 mL of 100 mg/L dye concentration, pH 9, and 0.1 g catalyst dose). The mixtures magnetically stirred in the dark at 500 rpm for 30 min to establish adsorption-desorption equilibrium. After that, the light was switched on, and aliquots of mixture were withdrawn every 15 min intervals over 90 min total reaction irradiation. Then centrifuged at 10,000 rpm for 10 min and filtered through 0.45 μm filters to remove any particulate matter. The total organic carbon content was measured using TOC-L analyzer (Shimadzu Corporation, Kyoto, Japan) equipped with non-dispersive infrared (NDIR) detector. The TOC removal efficiency was assessed using the following equation (Eq. 5).

5 $$ \:TOC\left( \% \right) = \:\frac{{TOC_{0} - TOC_{{f\:}} }}{{TOC_{0} }}\:x\:100 $$

Where, TOC_0_ and TOC_f_ are the initial and final total organic carbon concentration (mg C/L), respectively.

## Results and discussion

### Characterizations

#### Surface area (S_BET_)

**Fig. 2:**
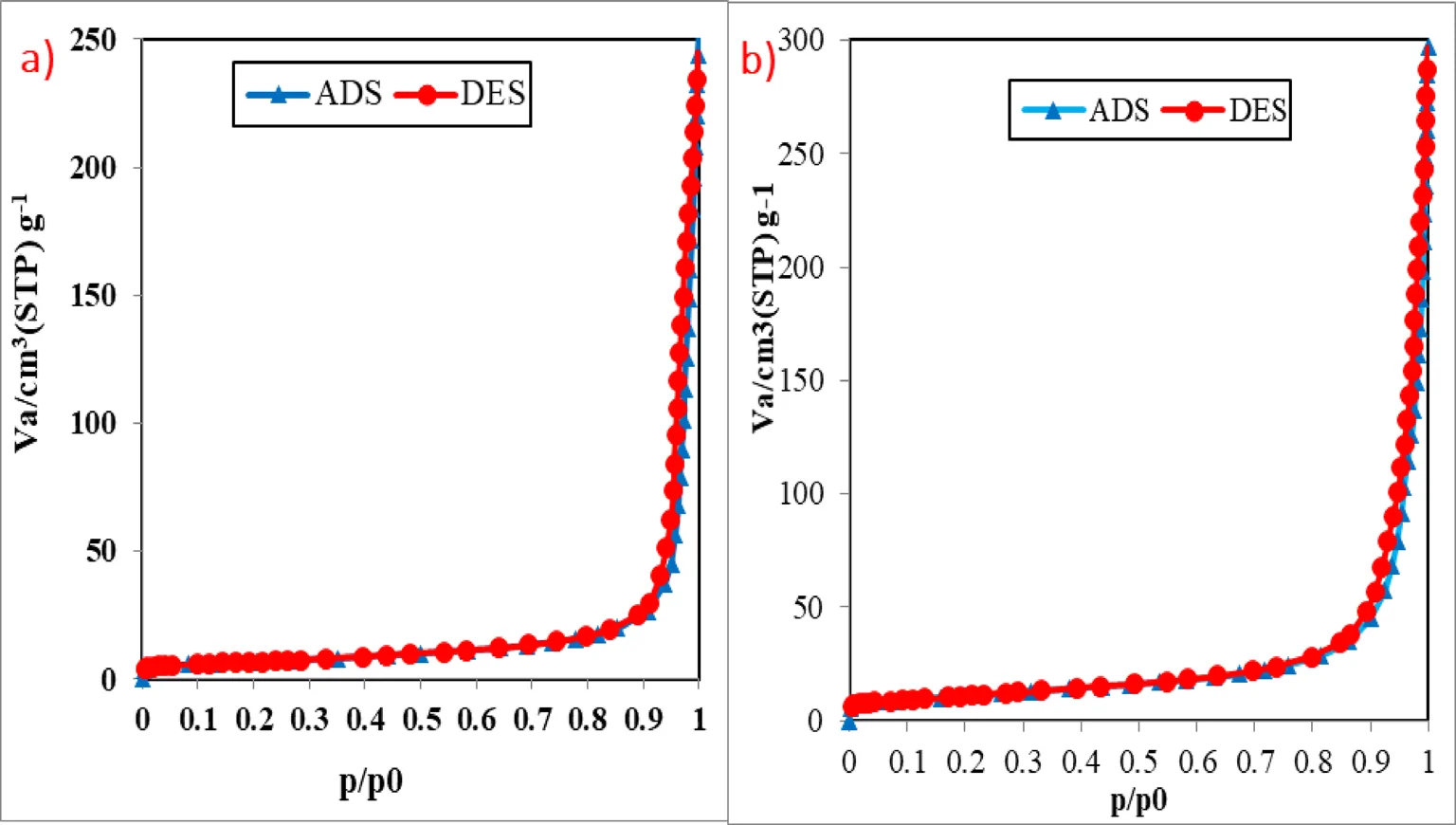
Nitrogen adsorption-desorption isotherms of **a**) chemically synthesized NiO/CoO and **b**) biogenically synthesized NiO/CoO@CM.

At low relative pressures, the produced chemical and green NiO/CoO nanocomposite had a minor increase in adsorption; however, as the pressure increased, a rapid rise was seen, exhibiting a classic type IV isotherm (Fig. 2a & b). This behavior confirms the presence of mesopores in the synthesized catalysts, particularly with a narrow hysteresis loop in the P/P₀ range of 0.5–1.0^38^. Moreover, the pore size distribution showed a limited range of pores smaller than 2.0 nm (Table 1), revealing that the produced catalysts had a clearly defined porous structure. The mesoporous structure facilitates the diffusion and transportation of dye molecules to active sites, associated with larger pore volume which provides more accessible surface area leading to increases of exposed active sites enhancing the adoption of dye molecules and accelerate the photocatalytic reaction^1^. NiO/CoO and NiO/CoO@CM nanocomposites have specific surface area values of 23.19 and 38.97 m^2^/g, respectively (Table 1). The notable increase of surface area for the biogenic NiO/CoO@CM suggests that the incorporation of algal extract enhances the material’s surface properties. These values are comparable to those reported by Abduljawad et al.^39^ for Ce@NiO@g-C_3_N_4_ nanocomposite (26.79 m^2^/g). Furthermore, the biogenic NiO/CoO@CM exhibited a larger average pore size and total pore volume (3.56 nm, 0.301 cm^3^/g) compared to the NiO/CoO (1.05 nm, 0.284 cm^3^/g) (Table 1).

**Table 1:** Surface area (S_BET_), total pore volume, pore diameter, and median pore diameters of NiO/CoO and NiO/CoO@CM nanocomposites.

Parameter	NiO/CoO	NiO/CoO@CM
Surface area BET, (m^2^/g)	23.19	38.97
Mean Pore diameter (nm)	48.90	30.91
Mean Pore Size (nm)	1.05	3.56
Total pore volume (cm^3^/g)	0.284	0.301
Median dimeter (nm)	76.00	76.63

#### Diffuse reflectance spectroscopy (DRS)

**Fig. 3:**
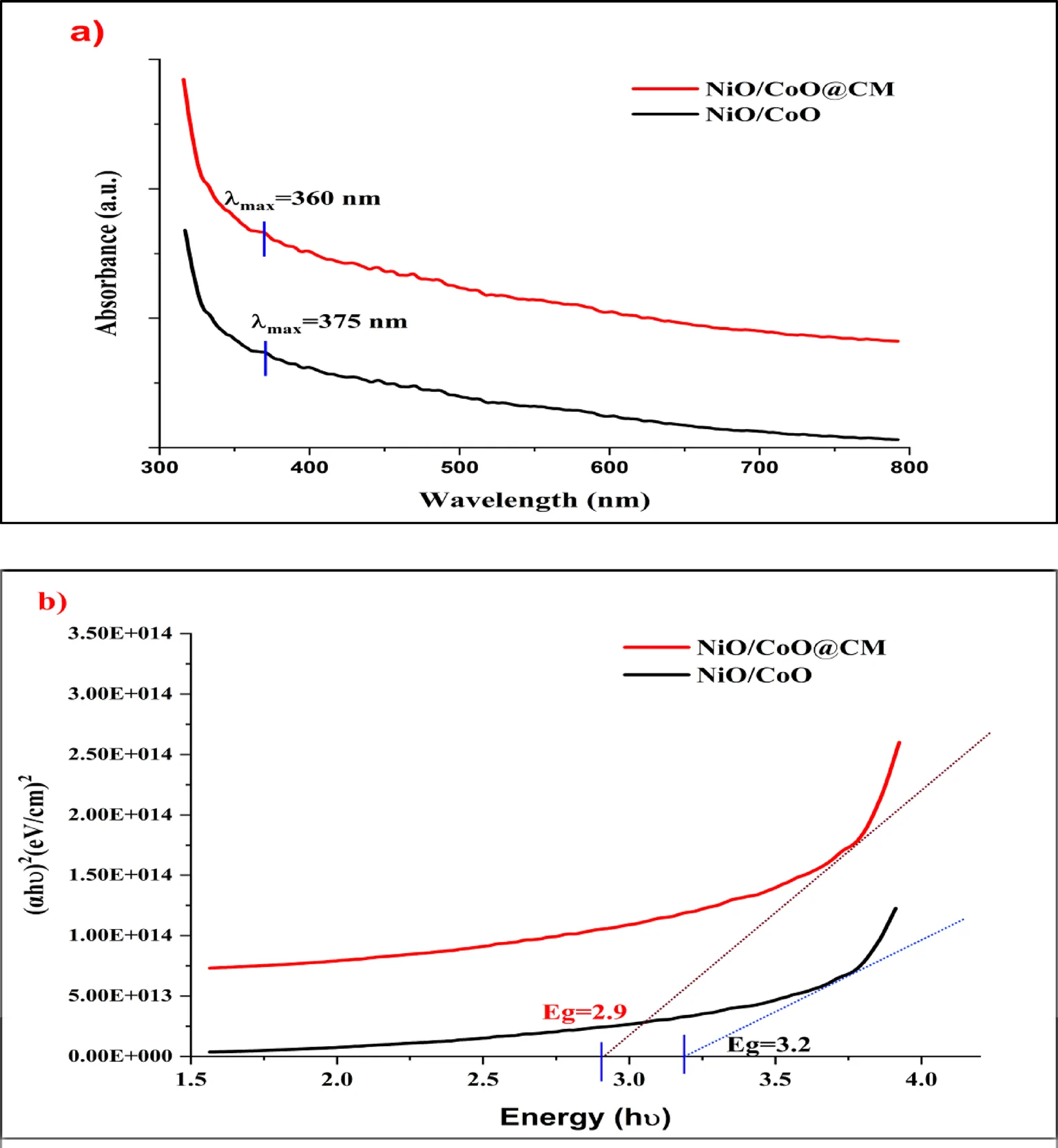
**a**) UV-Vis spectrum and **b**) Tauc’s graph of NiO/CoO and NiO/CoO@CM nanocomposites.

The optical properties of both synthesized catalysts were investigated using diffuse reflectance spectroscopy (DRS) (Fig. 3a). The chemically synthesized NiO/CoO displayed a maximum absorption peak at 375 nm in the UV region, whereas the biogenic NiO/CoO@CM exhibited a slight blue shift to 360 nm. This shift is attributed to the integration of bioactive gradients of algal extraction with metal oxide matrix^40^. Optical band gap energy (Eg) of both catalysts was estimated from linear extrapolation of the Tauc plots using Eq. (1), assuming a direct allowed electronic transition. The biogenically synthesized NiO/CoO@CM showed a lower band gap (2.9 eV) compared to that of NiO/CoO (3.2 eV) (Fig. 3b). The photodegradation efficiency of NiO/CoO@CM is improved by this decrease in band gap energy caused by bio-mediated intermediate states. High light absorption and improved charge carrier production are made possible by the decreasing band gap^40^. Furthermore, the integration of carbon in biogenic NiO/CoO@CM not only facilitates the absorption of light but also increasing the photodegradation efficiency, making it more effective for degrading hazardous organic compounds compared to unmodified NiO/CoO^41^.

#### Morphological properties and elemental analysis (SEM-EDX)

**Fig. 4:**
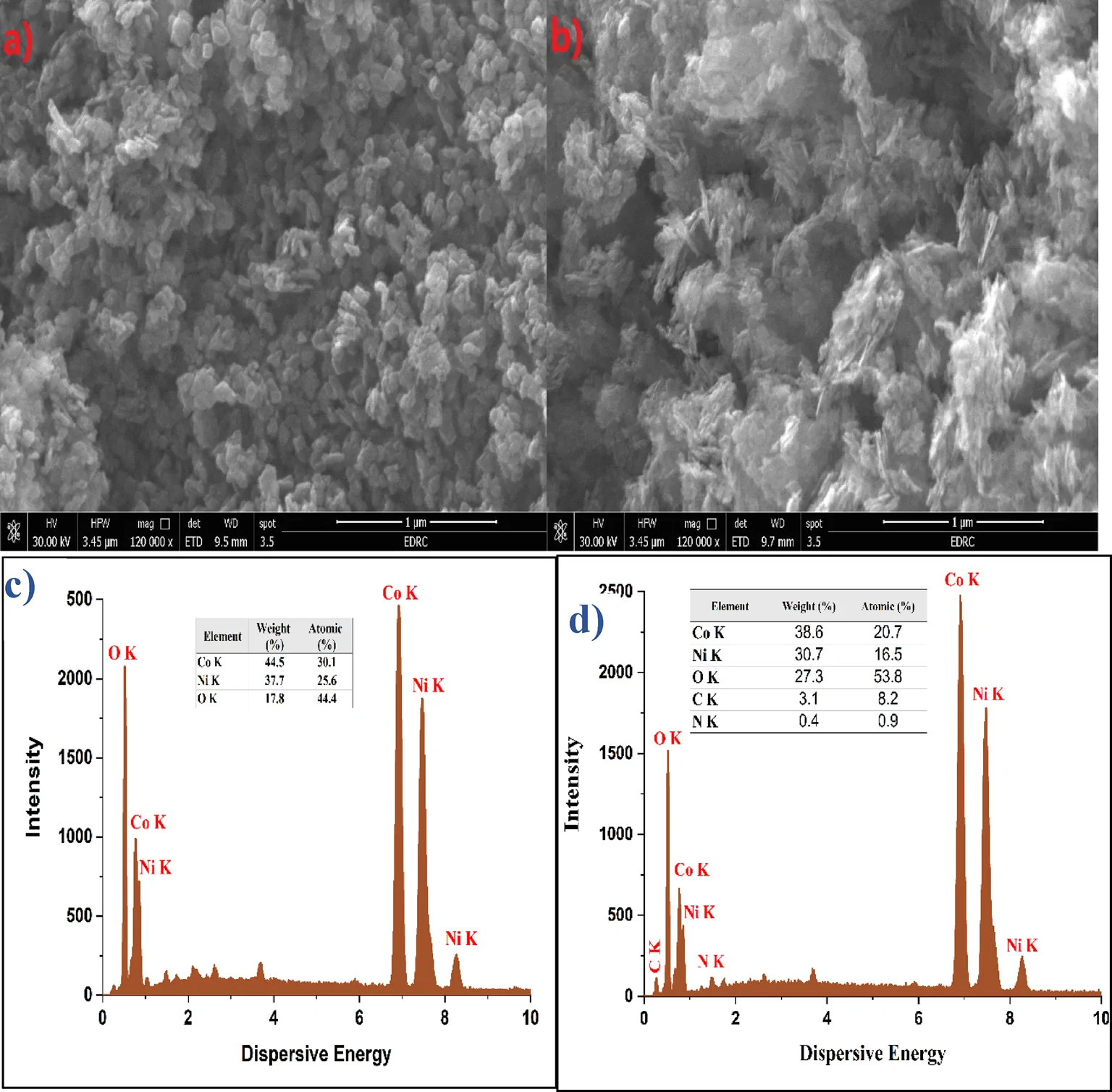
SEM image of **a**) chemically synthesised NiO/CoO, **b**) biogenic NiO/CoO@CM, **c**) EDX spectra of chemical NiO/CoO and **d**) biogenic NiO/CoO@CM,

The morphological features of NiO/CoO (Fig. 4a) showed irregularly shaped, tightly packed, and highly aggregated nanoparticles forming a rough, porous surface^40^. The particles showed clustered nanograins with cauliflower-like structure, revealing strong inter-particle interaction^42^. The nanoparticles were within the nanometer size range, with no obvious cracks or voids, suggesting a uniform distribution. In contrast, the SEM images of biogenic NiO/CoO@CM (Fig. 4b) exhibited a less compact and more loosely aggregated structure with flak-like structure^13^. The significantly difference in the morphological structures between chemical and biogenic nanoparticles confirms the influence of the organic bioactive compounds present in the extract during the green synthesis process^20^. The EDX spectrum of chemically prepared NiO/CoO (Fig. 4c) showed two major peaks for Co (44.5%) and Ni (37.7%), along with a minor peak for oxygen (17.8%), confirming the successful synthesis of a high-purity metal oxide nanocomposite. Conversely, the EDX spectrum of biogenic synthesized NiO/CoO@CM (Fig. 4d) showed slightly lower Ni (38.6%) and Co (30.7%), higher oxygen content (27.3%) and the presence of minor percentage of carbon (3.1%) and nitrogen (0.4%) peaks, which can be attributed to integration of bio-organic compounds from cyanobacterial extract onto the nanoparticle surface.

#### TEM analysis

**Fig. 5:**
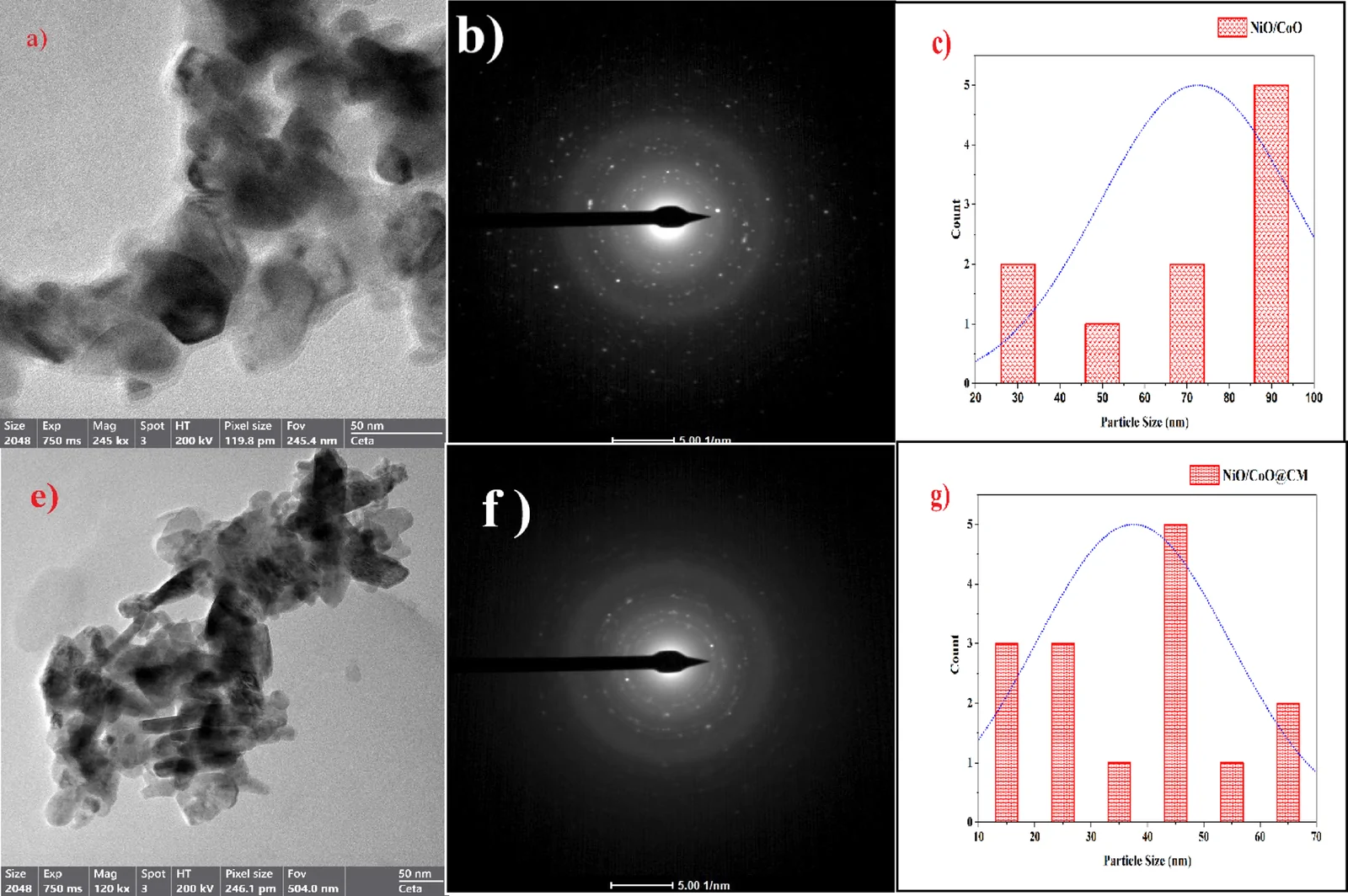
(**a**,** e**) TEM images, (**b, f**) selected area electron field (SAED) pattern and (**c, g**) average particle size distribution of histogram of NiO/CoO and NiO/CoO@CM respectively.

The TEM image of chemically synthesized NiO/CoO nanocomposite exhibits nearly spherical particles with relatively uniform shape and well-defined bounders (Fig. 5a). The corresponding selected area electron diffraction (SAED) shows a series of distinct concentric diffraction rings, confirming the polycrystalline nature of the material and indexing to the lattice planes of CoO and NiO phases, indicating the successful preparation of mixed metal oxides (Fig. 5b)^20^. The particle size distribution, performed from TEM micrograph, ranging from 36.4 to 95.7 nm with an average size of 52.5 nm (Fig. 5c). In contrast, the biogenically synthesized NiO/CoO@CM (Fig. 5e) shows irregular, elongated rod-like and flake-like nanostructures with higher particle aggregation with less uniformly distribution, which attributed to the capping and stabilizing effect of bio-compounds present in algal extract. Similarly, the SAED pattern of the green synthesized nanocomposite exhibits distinct diffraction rings, indicating the crystalline nature (Fig. 5f). The particle size is smaller than that of the chemically NiO/CoO, ranged between 11.0 and 65.1 nm with average of 33.5 nm (Fig. 5g).

#### X-Ray diffraction (XRD)

**Fig. 6:**
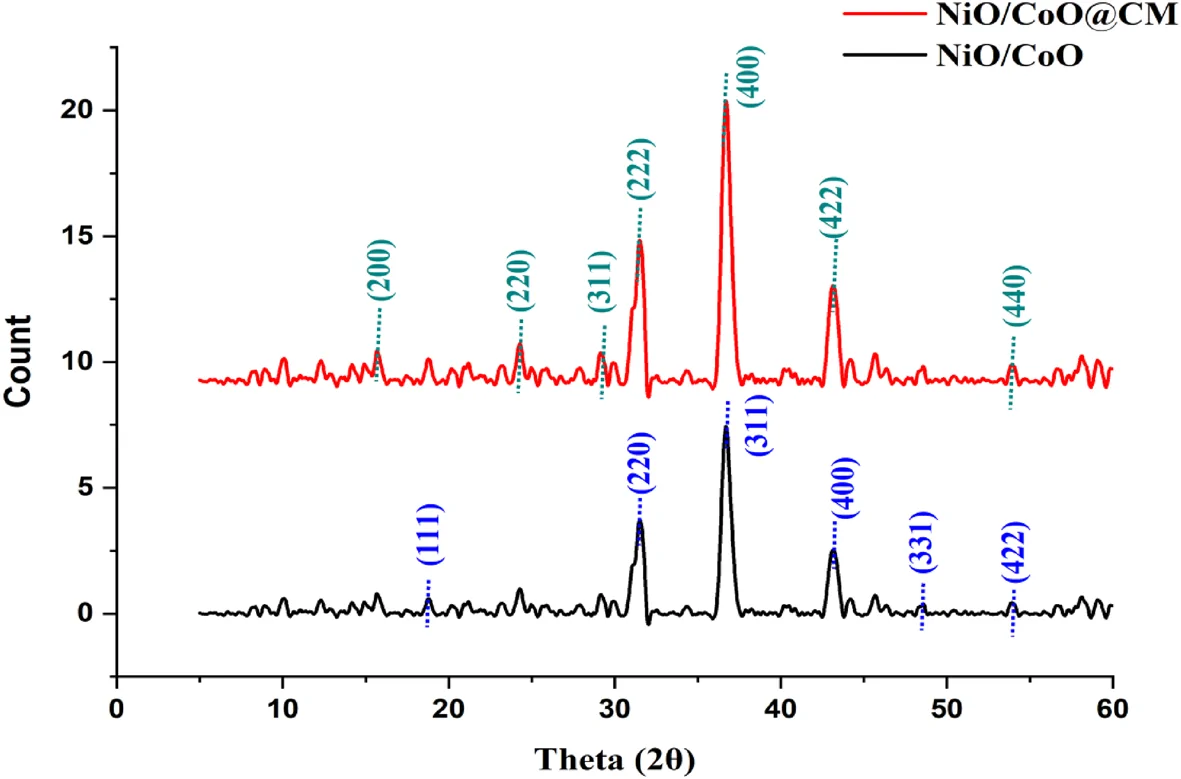
XRD pattern of chemically synthesized NiO/CoO and biogenic NiO/CoO@CM nanocomposites.

The phase composition and crystal structural of the chemically synthesized NiO/CoO nanocomposites were conducted in Figure (6). A prominent diffraction peaks at 2θ values of 18.92⁰, 31.15⁰, 36.70⁰ and 44.63⁰, which corresponding to (111), (220), (311) and (400) crystallographic planes, of cubic NiO (JCPDS cards No. 01-073-1702^37^. In addition, two peaks were observed at 48.89° and 55.43°, assigned to the (331) and (422) planes of cubic CoO phases within the nanocomposite^43^. The average crystallite size of NiO/CoO was calculated using Debye-Scherrer equation (Eq. 2), the crystallite size ranged from 22.26 to72.78 nm, with mean value of 37.79 nm, confirming the nanoscale dimensions of the prepared materials^44^. The coexistence of NiO and CoO crystalline phases together with CPS and TEM findings supports the successful formation of the NiO/CoO heterostructured nanocomposites.

The XRD pattern of the biogenically synthesized NiO/CoO@CM nanocomposite showed diffraction peaks with comparable to those of NiO/CoO, with slight shifts in the 2θ positions at 17.94⁰, 25.42⁰, 29.65⁰, 31.16⁰, 36.26⁰, 44.71⁰ and 52.07⁰ corresponding to (200), (220), (311), (222), (400), (422), and (440) planes, respectively which are indexed to the cubic phases of NiO and CoO (JCPDS card No. 00-022-1184). The appearance of additional reflections indexed to the (222), (422), and (440) supports the formation of NiO and CoO phases within the mixed mixed-metal oxide nanocomposite^13^. The average crystallite size of NiO/CoO@CM was relatively smaller than that of NiO/CoO, ranging from 8.31 to 50.72 nm with an average of 27.05 nm. This reduction in the particle size accompanied by increasing crystallinity, indicates that the cyanobacterial extract effectively controlled the morphology of the prepared catalysts and reduced their crystallite size through the diverse array of bioactive compounds present in the extract, such as polysaccharides, proteins, pigments and other secondary metabolites which act as capping agents. These biomolecules inhibit the overgrowth and prevent aggregation of nanoparticles leading to formation of smaller and more uniform crystallite with defined shapes^45^.

#### XPS analysis

**Fig. 7:**
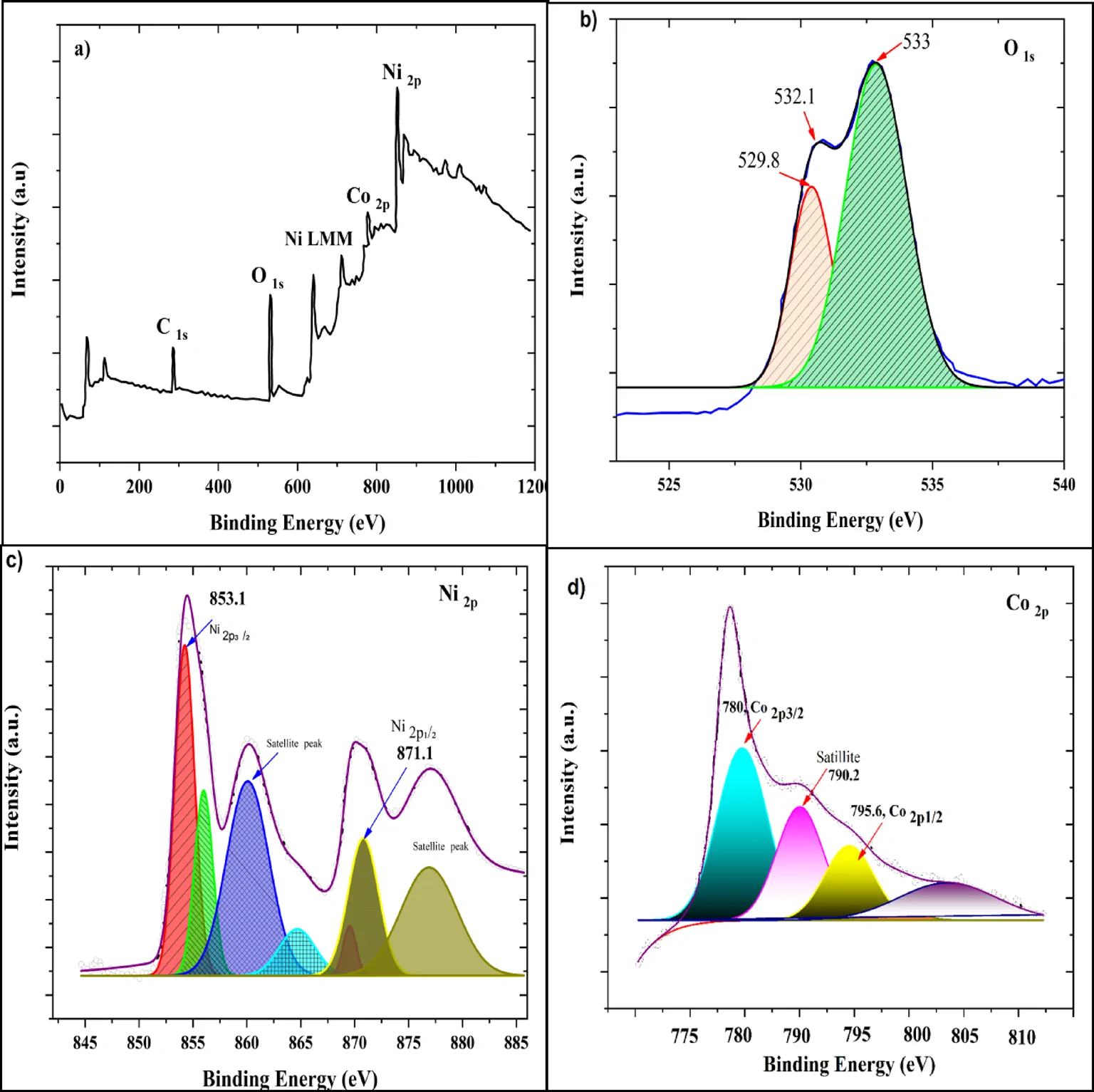
XPS analysis of NiO/CoO nanocomposite: **a**) survey spectrum, **b**) high resolution O 1s spectrum, **c**) Ni 2P spectrum and **d**) Co 2p spectrum.

The oxidation states and surface composition of the prepared NiO/CoO nanocomposites were examined using XPS. The survey spectrum of the catalyst revealed the presence of Ni, Co and O elements, along the C used as charge references (C 1s = 284.8 eV) (Fig. 7a). The high resolution O 1s spectrum was deconvoluted into three peaks (Fig. 7b), a dominant peak at 529.8 eV attributed to lattice oxygen (O^2−^) within the metal oxygen framework, a peak at 532.1 eV assigned to surface bound oxygen and hydroxyl groups, and a peak at 533.0 eV corresponding to adsorbed molecular water. The presence of such hydroxyl functionalities is known to enhance photocatalytic activity by promoting generation of reactive oxygen species^35^. The high resolution Ni 2p spectrum (Fig. 7c) showed two main peaks at 853.1 and 871.1 assigned for Ni 2p₃/₂ and Ni 2p₁/₂, respectively. Another tow prominent satellite peaks appeared at 861.3 eV and 879.0 eV confirming the presence of Ni2 + characteristic of NiO^46^. Similarly, Co 2p spectrum (Fig. 7d) showed two main peaks at 780.9 eV and 795.6 eV assigned to Co 2p₃/₂ and Co 2p₁/₂, respectively with two broad satellite peaks at 790.2 and 802.5 eV characteristic of the Co^2+^ oxidation state in CoO^20^.

#### FTIR analysis

**Fig. 8:**
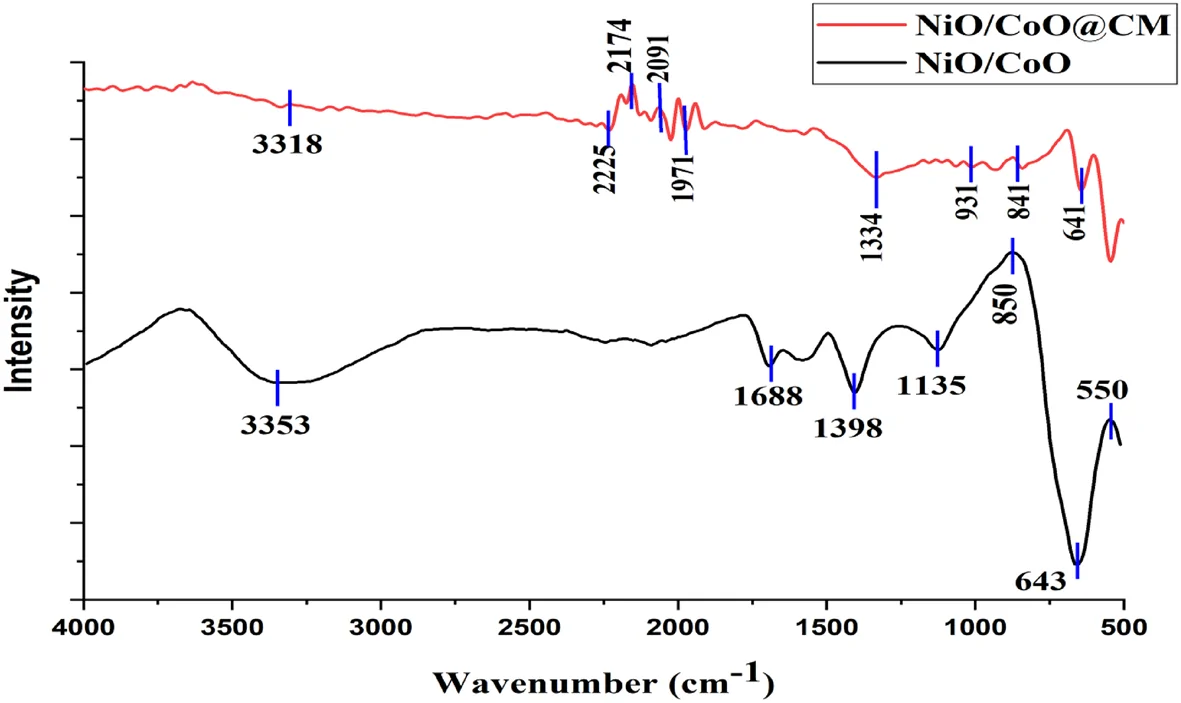
FTIR spectra of chemically synthesized NiO/CoO and biogenic NiO/CoO@CM nanocomposites.

Figure (8) displays the FTIR spectra of the chemically and biogenically prepared nanocomposites. The chemically synthesized NiO/CoO catalyst showed several characteristics absorption bands. A broad band at 3353 cm^− 1^ was assigned to O-H stretching vibration of surface hydroxyl group, and a band at 1688 cm^− 1^ corresponded to H-O-H bending vibration of adsorbed water molecule^47^. The bands appeared at 1398 cm^− 1^ and 1135 cm^− 1^ are typically attributed to C-O stretching vibrations for carbonates^48^, while the peak at 850 cm^− 1^ is assigned to out-of-plane C-H bending vibrations. Notably, two intense bands observed at 643 cm^− 1^ and 550 cm^− 1^ are assigned to metal-oxygen bond (Ni-O and Co-O) stretching vibrations, confirming formation of NiO and CoO structure^20^.

The FTIR spectrum of biogenically synthesized NiO/CoO@CM showed a similar functional groups with slightly shifts in band positions compared to the chemically synthesized materials. The O-H stretching vibration band appeared at 3318 cm^− 1^, while Ni-O and Co-O vibrational bands were observed at 641 cm^− 1^ and 551 cm^− 1^ (Table 2). Multiple additional new peaks were observed in the range of 2225 –1971 cm^−^, which are assigned mainly to carbonyl groups (C = O), and atmospheric CO_2_^21^. Peaks at 931 cm^− 1^ and 841 cm^− 1^ are assigned to C–O and C–N stretching vibrations, indicating the presence of polysaccharides and proteins from the cyanobacterial extract^21^. Collectively, FTIR spectra reveal that the cyanobacterial extract introduces additional functional groups such as nitriles, polysaccharides and amides, which can enhance the surface functionality, dispersion, and photocatalytic performance of the biogenic nanocomposite.

**Table 2:** Key FTIR Peaks and their assignments.

Wavenumber (cm⁻¹)	Assignment	Catalysts
3353, 3318	O–H stretching (hydroxyl/water)	NiO/CoO and NiO/CoO@CM
2225–1971	C = O stretching (organics)	NiO/CoO@CM
1688	H–O–H bending (water)	NiO/CoO
1398, 1334	C–O, C–N stretching	NiO/CoO and NiO/CoO@CM
1135, 931, 841	C–O, C–N, polysaccharides	NiO/CoO@CM
850	O–H/C–H bending	NiO/CoO
643, 641, 551	Ni–O, Co–O stretching	NiO/CoO and NiO/CoO@CM

### Photocatalytic activity

#### Effect of pH

**Fig. 9:**
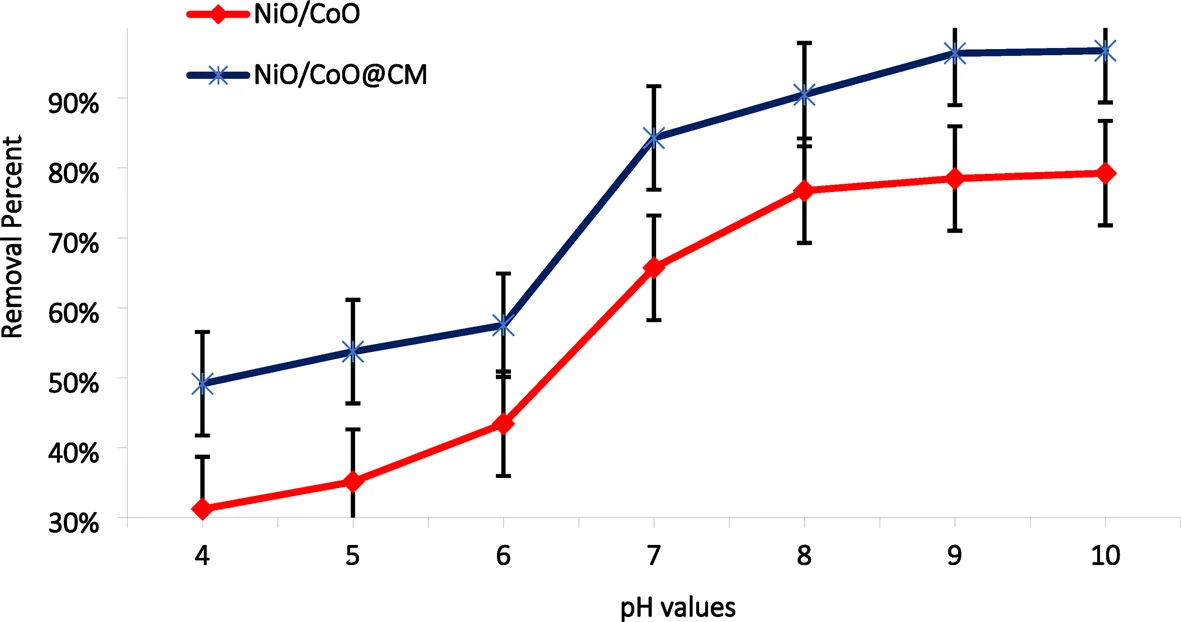
Influence of solution pH on the photodegradation efficiency of CV dye using NiO/CoO and NiO/CoO@CM nanocomposites.

The influence of solution pH on the degradation efficiency of crystal violet (CV) dye was investigated over the pH range of pH 4 to 10 using both synthesized catalysts (Fig. 9). The correlation between pH and adsorption efficiency is strongly governed by the pH at which the surface of the molecule has no charge (pH_PZC_). Below the pH_PZC_, the catalyst surface becomes positively charged, leading to electrostatic repulsion with the cationic CV dye molecules, thus lowering the removal efficiency^21^. Conversely, above pH_PZC_, the catalyst surface gets a negative charge, which increases electrostatic attraction with the positively charged dye molecules, thereby promoting higher removal efficiency^49^.

At acidic conditions (pH 4–6), both catalysts exhibited relatively poor removal efficiencies, with NiO/CoO achieving 30–40% while biogenic NiO/CoO@CM catalyst achieving 50–60%. This lower removal performance at low pH is attributed to the proton-rich (H^+^) medium which imparts a positive charge to the catalyst surface, leading to electrostatic repulsion with cationic dye^50^. As the pH was raised to neutral and alkaline levels (pH 7–10), a noticeable improvement in removal efficiency was observed for both catalysts. At pH 9, the maximum degradation efficiency reached 96.5% for NiO/CoO@CM and 79.2% for NiO/CoO. The increase generation of hydroxyl radical (•OH) associated with elevated pH, giving the catalyst a negative charge and facilitates stronger electrostatic interaction with cationic CV molecules^51^. The superior performance of the biogenic NiO/CoO@CM catalyst is attributed to its enhanced surface properties, such as a larger surface area, smaller particle size, and the presence of more active sites introduced by bio-functional groups from cyanobacterial extract. These findings are in good agreement with reported by Ramzan et al.^52^, who achieved 96.5% removal at pH 9 using sulfur-doped NiCr_2_O_4_ nanocomposites, and Jeevarathinam and Asharani^51^, who found 96% degradation efficacy at pH 10 using g-C_3_N_4_/CuO nanocomposites, are both consistent with these findings.

#### Influence of contact time

**Fig. 10:**
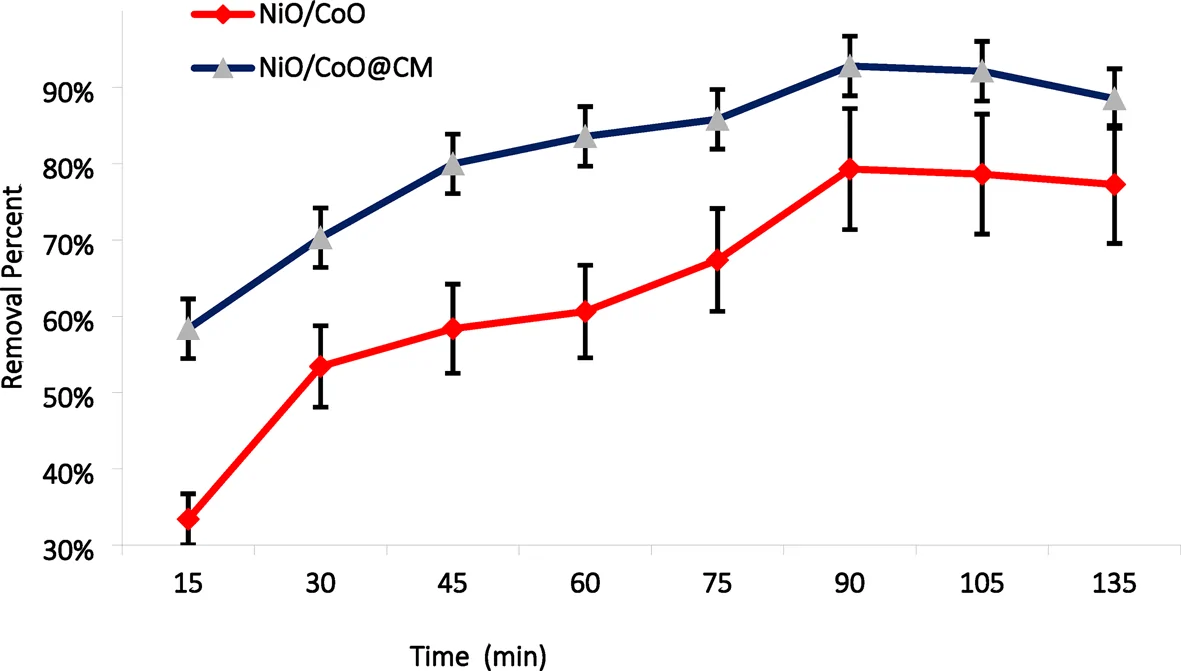
Influence of contact time (min.) on the photodegradation efficiency of CV dye using NiO/CoO and NiO/CoO@CM nanocomposites.

The degradation efficiency of crystal violet (CV) dye using both catalysts was evaluated over a contact time ranging from 15 to 135 min to determine the optimal time for achieving maximum removal efficiency (Fig. 10). A rapid increase in degradation efficiency was observed for both catalysts during the initial time phase (15–60 min) reached 83.3% for biogenic NiO/CoO@CM and 60.63% for NiO/CoO. This rapid rate is attributed to the high availability of active sites on the catalyst surface during early reaction stages^3^. The degradation rate gradually decreased as the contact time increased from 60 to 135 min, indicating saturation of active sites^53^. The chemically synthesized NiO/CoO achieving 79.3% degradation of CV and biogenic NiO/CoO@CM achieving 92.8%. Owing to the bio-functional groups added by the cyanobacterial extract, the biogenic NiO/CoO@CM showed superior degradation performance, which resulted in greater interaction between the dye molecules and the catalyst surface, enhanced electron-hole separation, and higher photocatalytic reactivity^22^. These findings align with previously reported studies, such as Yelpale et al.^54^, achieved 99% removal of CV dye using ZnO within 150 min, Manikandan et al.^11^, who reported 99.9 degradation in 60 min using rGO/ZnCo_2_O_4_ nanocomposite and Dhanabal and Priya^55^, who achieved 98.2% removal of CV dye in 90 min using Sr-ZnO/activated carbon nano-needles.

#### Effect of photocatalyst dose

**Fig. 11:**
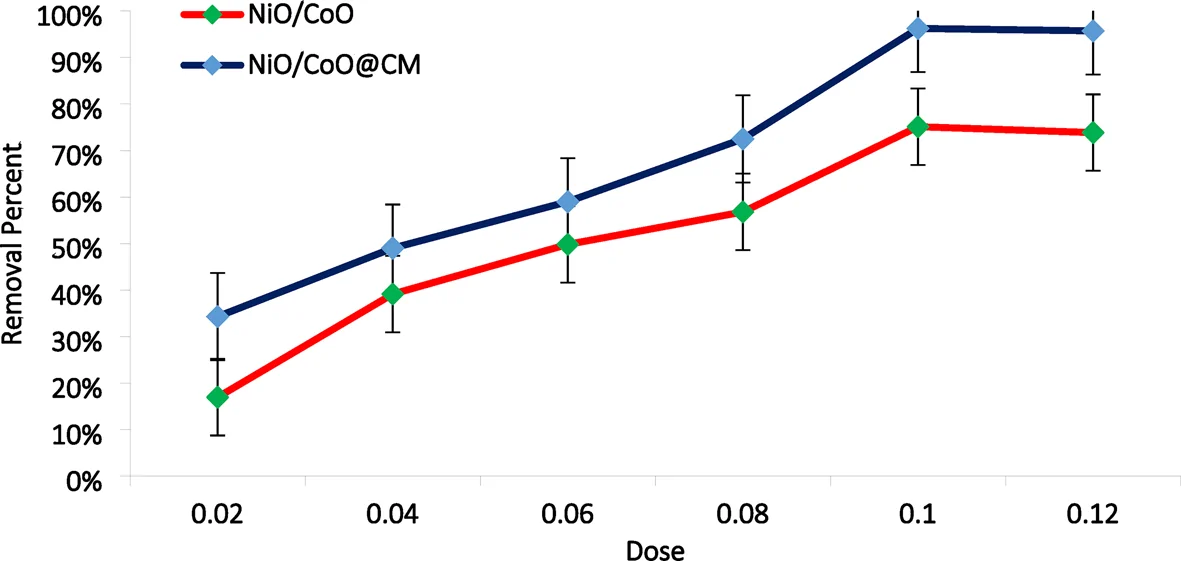
Influence of catalyst dosage on the photodegradation efficiency of CV dye using NiO/CoO and NiO/CoO@CM nanocomposites.

To investigate the influence of catalyst dosage on the CV removal efficiency, the catalyst amounts ranging from 0.02 to 0.12 g per 50 mL of dye solution was used. A significant rise in the degradation rate was noted for both catalysts as the catalyst dose increased, which is attributed to the enhanced availability of active surface sites promoting dye absorption and subsequent increase degradation reactions^56^. The biogenic NiO/CoO@CM achieved higher removal efficiency of 96.3% at a dosage of 0.1 g/50 mL, while the chemically synthesized NiO/CoO exhibited lower efficiency of 75.1% under the same conditions (Fig. 11). The marked improvement in degradation efficiency of the biogenic NiO/CoO@CM is explained by its larger specific surface area (38.97 m²/g) compared to NiO/CoO, which provides a greater active sites numbers. Additionally, the bio-functional groups introduced by the cyanobacterial extract. These groups reduce particle clumping, enhance dispersion, and offer extra active sites for degradation processes^34^. These results are in good agreement with previous studies; Samuel et al.^31^ who reported 98% removal of acid blue 74 dye using 0.6 g/L of CoO nanoparticle synthesized using Jumbo Muscadine (*Vitis rotundifolia*) extract, and Rong et al.^57^ who achieved 100% removal of CV dye using 0.5 g/L of RuO_2_/g-C_3_N_4_ nanocomposites.

#### Effect of initial dye concentration

**Fig. 12:**
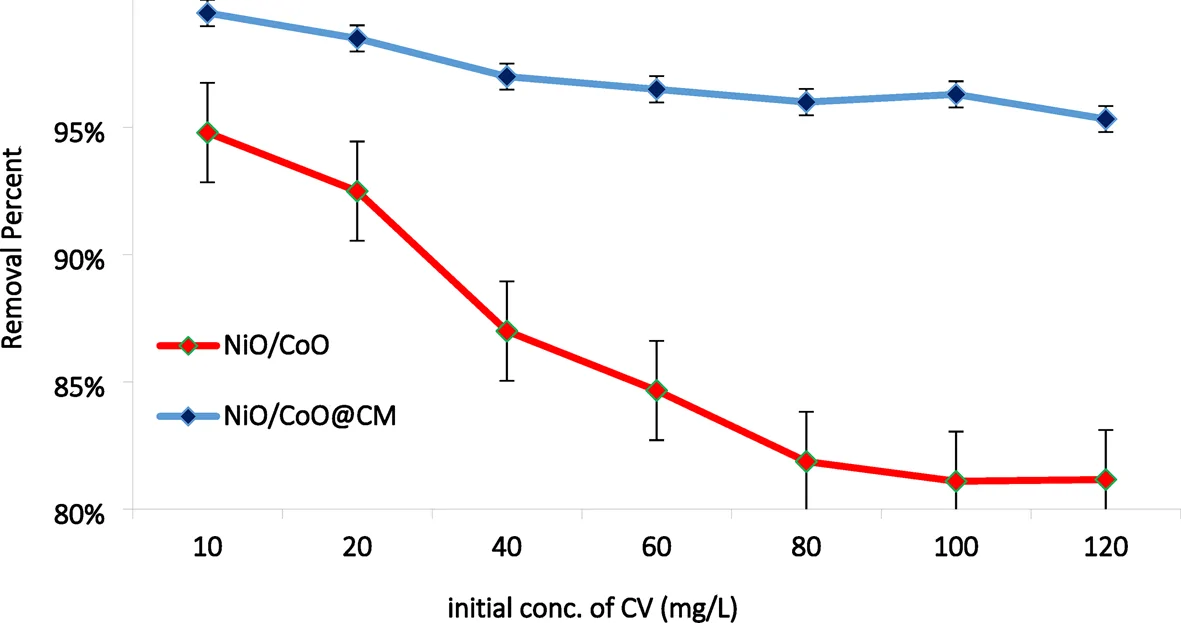
Influence of initial CV dye concentration (mg/L) on the photodegradation efficiency using NiO/CoO and NiO/CoO@C M nanocomposites.

The effect of the initial concentration of crystal violet (CV) dye (20–120 mg/L) on the removal efficiency of both synthesized catalysts is shown in Figure (12). A noticeable decrease in removal efficiency was observed at lower dye concentrations (20–80 mg/L), from 94.8 to 81.88% for NiO/CoO and from 99.5 to 96.5% for NiO/CoO@CM, which reflects the greater availability of active sites on the surface of catalyst for interaction with dye molecules^51^. At equilibrium, the removal efficiencies of CV were 81.1% for NiO/CoO and 96.3% for NiO/CoO@CM. However, a further increase in the initial concentration of CV dye beyond 100 mg/L resulted in slowdown of the reaction rate. This is mainly attributed to the saturation of active sites on the catalyst surface which limits adsorption capacity thus reduces the available surface area for photocatalytic reaction and decreasing light penetration into the solution^1^.

### Total organic carbon removal (TOC%)

**Fig. 13:**
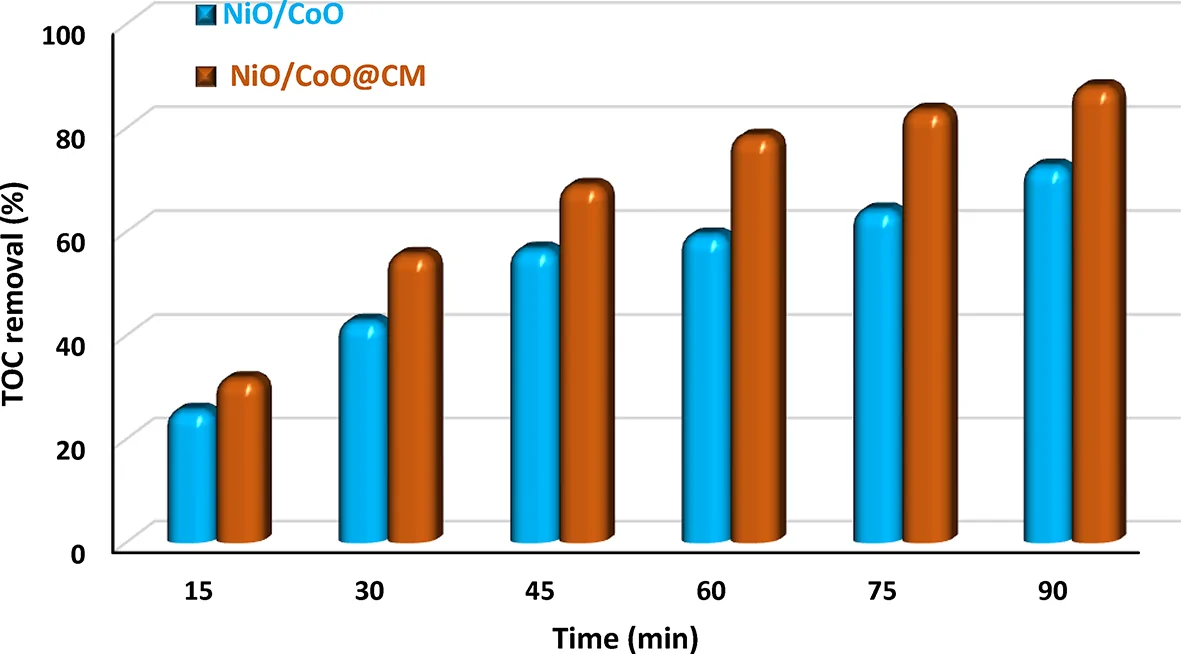
TOC removal efficiencies of NiO/CoO and NiO/CoO@CM catalyst fro crystal violet degradation.

The biogenic NiO/CoO@CM catalyst exhibited a remarkable TOC removal of 87.9% for crystal violet within 90 min, compared to 72.6% for the chemically synthesized NiO/CoO under the same conditions (Fig. 13). Although the corresponding decolorization efficiencies were slightly higher (79.3% for NiO/CoO and 96.5% for NiO/CoO@CM), the relatively lower TOC removal reveals the possible formation of colorless organic intermediates by-products during the photocatalytic process, suggesting that the complete mineralization proceeds through several steps before final formation of H_2_O and CO_2_^58^. The biogenic catalyst exhibited a remarkable accelerated mineralization kinetics, achieving 78.5% TOC removal within first 60 min compared with 59.4% for NiO/CoO, which attributed to enhanced physiochemical characteristics of the biogenic nanocomposite, including higher surface area (38.97 m^2^/g), narrower band gap (2.9 eV) reduced crystallite size (27.05 nm) and abundant surface adsorbed bioactive functionalities^59^.

### Reusability and stability experiment

**Fig. 14:**
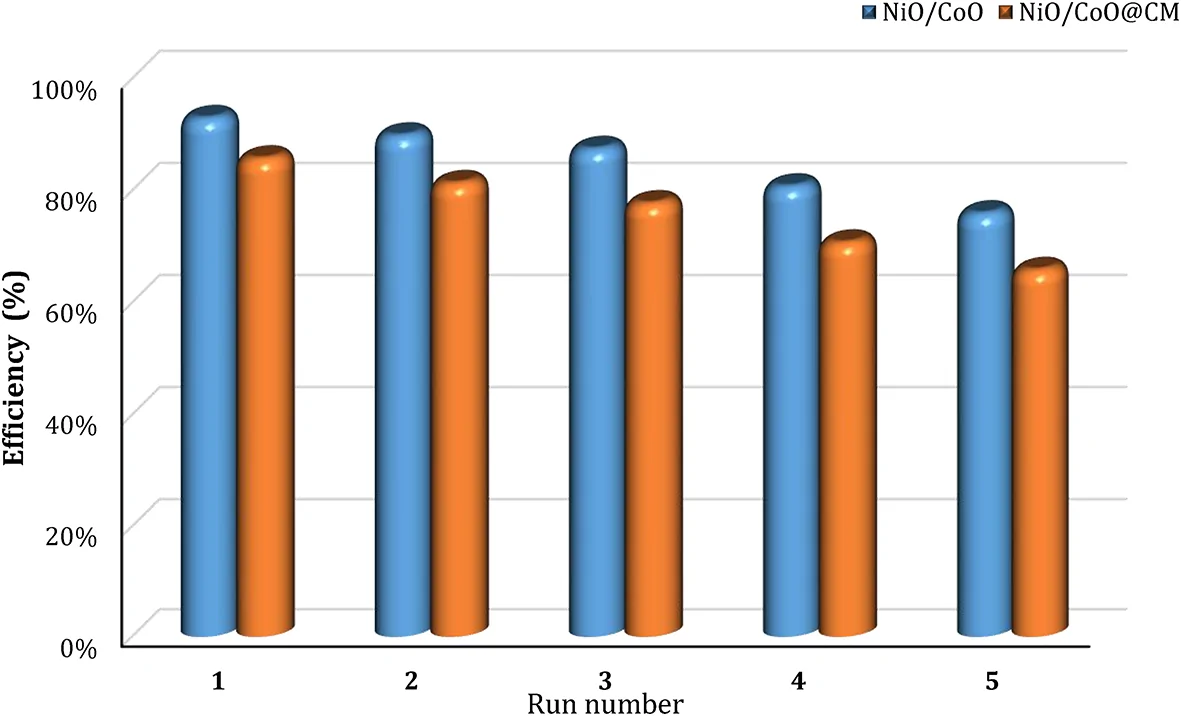
Reusability experiment of NiO/CoO and NiO/CoO@CM catalysts across five successive cycles.

The reusability and stability of the synthesized catalysts were estimated over five successive cycles (Fig. 14). Both catalysts showed a slight decrease in photocatalytic efficiency, which owing to partial catalyst loss and the accumulation of the by-product intermediates on the active sites^60^. The biogenic NiO/CoO@CM showed high stability, with slight decrease of degradation from 92.8% (Cycle 1) to 75.8% (Cycle 5). While, NiO/CoO showed decline from 80.5% to 65.7%, confirming a faster deactivation rate which attributed to particles aggregation leading to diminished suspension stability and reduced active site availability^61^.

### Mechanism of photodegradation of crystal violet

**Fig. 15:**
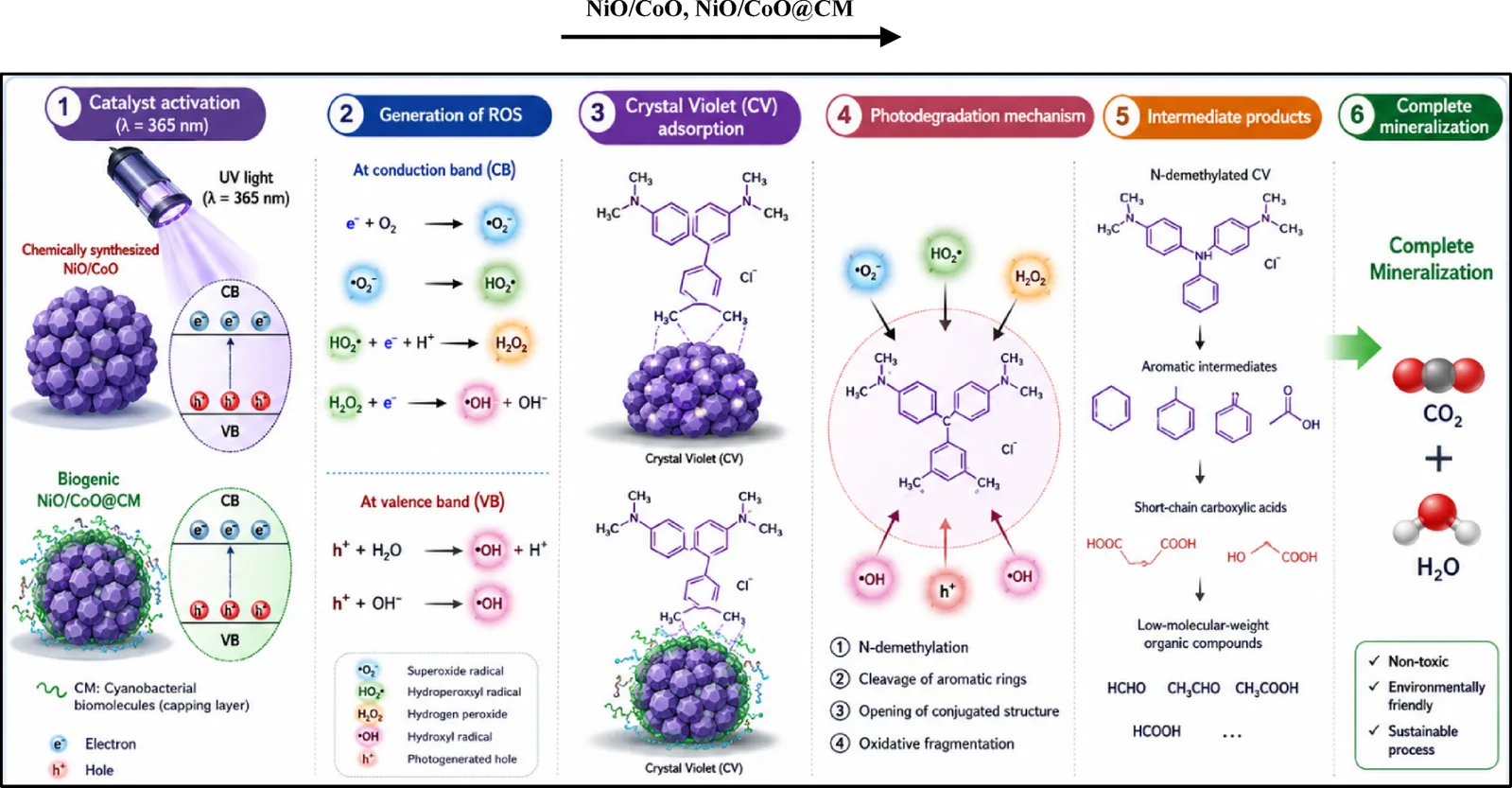
Schematic diagram illustrates the photodegradation mechanism of CV over NiO/CoO and NiO/CoO@Cm.

Figure (15) illustrates the proposed photocatalytic mechanism for CV degradation over both synthesized catalysts. Upon UV irradiation (l < 365), the catalyst particles are photoexcited, promoting electrons transition from the valence band (VB) to conduction band (CB) leaving positive holes (h+) in the VB^54^.

$$ NiO/CoO + hv~ \to ~NiO/CoO\left( {e_{{CB}}^{ - } + ~h_{{VB}}^{ + } } \right) $$

These highly charge carriers migrate to the catalyst surface, initiating the ROS generation. The electrons in conduction bands ($\:{e}_{CB}^{-}$) reduce dissolved molecular oxygen forming superoxide radicals ($\:{O}_{2}^{-}),\:$which are then converted to hydrogen peroxide (H_2_O_2_) and further cleaved to hydroxyl radicals (•OH). Additionally, the valence band holes ($\:{h}_{VB}^{+}$) oxidize the adsorbed water or hydroxyl ions yielding more hydroxyl radicals ((•OH) (Mohamed et al.^12^.

$$ e_{{CB}}^{ - } + ~O_{2} \to ~ \cdot O_{2}^{ - } $$

$$ O_{2}^{ - } + h^{ + } \to \cdot OH_{2} $$

$$ 2 \cdot OH_{2} \to ~H_{2} O_{{2~}} + ~O_{2} $$

$$ H_{2} O_{{2~}} + ~e_{{CB}}^{ - } ~ \to \cdot OH + ~OH^{ - } $$

$$ h_{{VB}}^{ + } + ~H_{2} O_{{~~}} \to \cdot OH + ~H^{ + } $$

These reactive oxygen species (•OH and $\:{O}_{2}^{-}$) attack the triphenylmethane ring of CV dye, break the conjugated structure and yielding an open N-demethylation-chromophore intermediate, followed by progressive fragmentation of the dye into various low molecular weight intermediates, till achieving final mineralization into carbon dioxide and water molecules^62^.  

$$ \:C_{{25}} H_{{30}} ClN_{3} + \:OH + \:H^{ + } \mathop \to \limits^{{NiO/C_{o} O,NiO/C_{o} O@CM}} CO_{2} + H_{2} O + {\mathrm{inorganic}}\:{\mathrm{ions}} $$

### Isothermal studies

Different isothermal models were applied to assess the crystal violet adsorption behavior onto the surfaces of NiO/CoO and NiO/CoO@CM nanoparticles using Langmuir and Freundlich isotherm models. The linear form of the Langmuir equation (Eq. 4) is expressed as;

4 $$\:\frac{1}{{q_{e} }} = \frac{1}{{q_{{max}} }} + \frac{1}{{K_{L} q_{{max}} }} \cdot \frac{1}{{c_{e} }} $$

The dimensionless Langmuir constant (R_L_) was calculated using the following equation.

5 $$ R_{L} = {{~}}\frac{1}{{1 + K_{L} C_{e} }} $$

where: q_max_ (mg/g) represent the maximum adsorption capacity; q_e_ (mg/g) represent the amount of CV adsorbed at the equilibrium; C_e_ (mgL^− 1^) represent the equilibrium concentration; and K_L_ represent Langmuir constant (L/mg).

The Freundlich isotherm model is expressed by the following linear form.

6 $$\:log{q}_{e}={logK}_{f}+{\frac{1}{n}logC}_{e}^{}$$

Where; K_f_ is and *n* are Freundlich constants for adsorption capacity and intensity respectively.

**Fig. 16:**
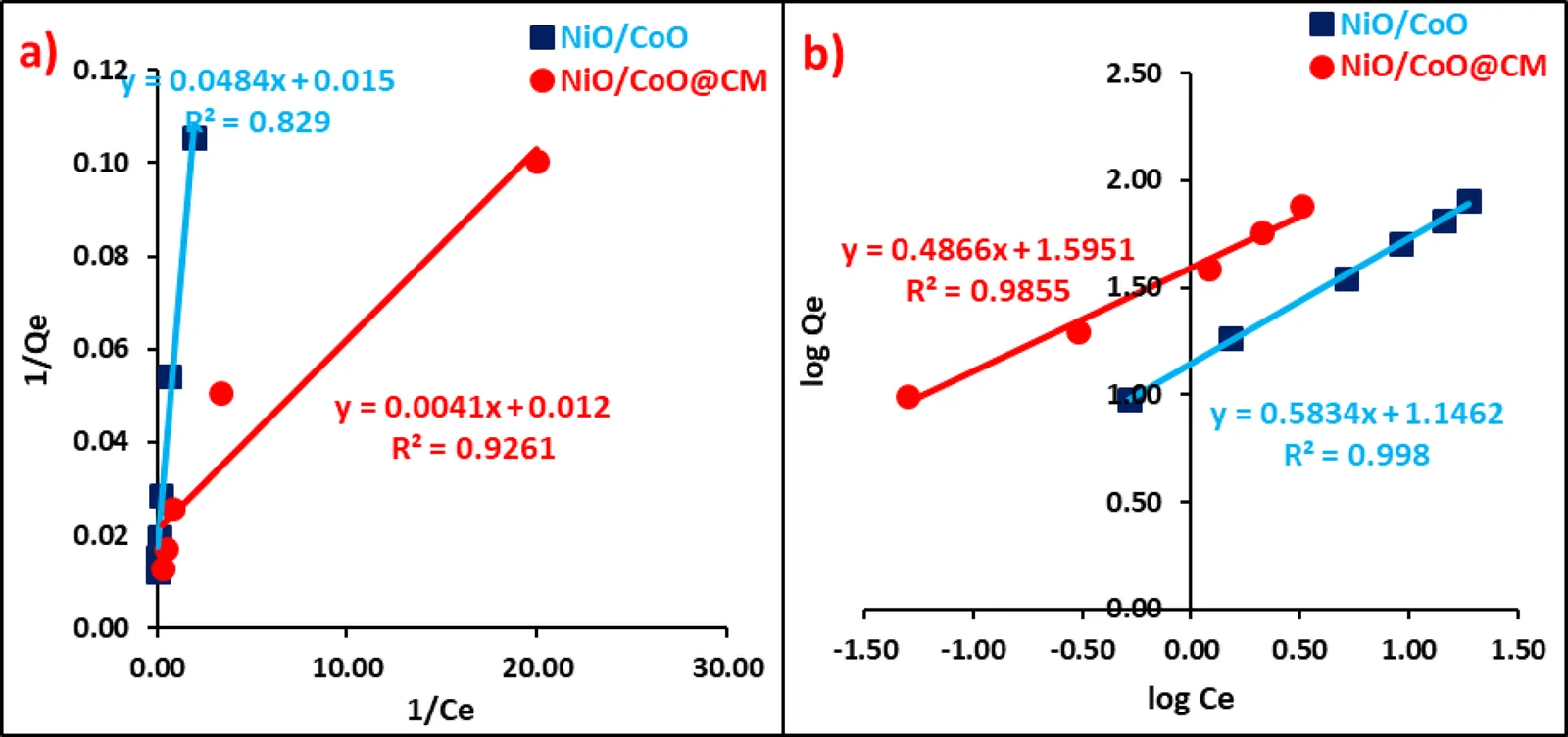
Isotherm models for CV dye adsorption dye onto NiO/CoO and NiO/CoO@CM surfaces (**a**) Langmuir model and (**b**) Freundlich model.

The key parameters derived from both isotherm models are summarized presented in Table (3) and Figure (16 a&b). The Langmuir model showed moderate correlation coefficient (R^2^ = 0.83 for NiO/CoO and 0.93 for NiO/CoO@CM), which were notably lower than those obtained from the Freundlich model (R^2^ >0.98). The higher K_L_ value for NiO/CoO@CM (5.12 L/mg) indicates a stronger binding affinity compared for NiO/CoO (0.31 L/mg). Additionally, the R_L_ values for both catalysts fell within the range of 0 to 1, revealing favorable adsorption behavior^63^. However, Freundlich model provided a better fit for both catalysts, with R^2^ values exceeding 0.98. The biogenic NiO/CoO@CM showed a higher K_f_ (39.36 mg/g) compared to NiO/CoO (14.0 mg/g) suggesting a greater adsorption capacity. Furthermore, the *n* values for both catalysts (1.71 for NiO/CoO and 2.06 NiO/CoO@CM) were greater than 1, revealing favorable adsorption conditions and strong interaction between the catalyst surfaces and dye molecules^1^.

**Table 3:** Isothermal key parameters for CV dye adsorption dye onto NiO/CoO and NiO/CoO@CM surfaces.

Model of Isotherm	Langmuir Isotherm				Freundlich Isotherm		
Catalyst	K_L_. (L/mg)	q_max_. (mg/g)	*R*_L_	*R* ^2^	K_f_ (mg/g)	*n*	*R* ^2^
NiO/CoO	0.31	66.67	0.03	0.83	14.00	1.71	0.998
NiO/CoO@CM	5.12	82.62	0.02	0.93	39.36	2.06	0.986

### Kinetic studies

Two commonly kinetic models were applied to demonstrate the adsorption behavior of CV dye onto the surface of both catalysts: the pseudo-first-order (PFO) and pseudo-second-order models (PSO). The linear form of PFO and PSO kinetic equations is expressed as follow;

7 $$\:\mathrm{log}\:({q}_{e}-{q}_{t})=log{q}_{e}-\:\frac{{k}_{1}}{0.203}\:t$$

8 $$\:\frac{t}{{q}_{t}}=\:\frac{1}{{k}_{2}{q}_{e}^{2}}+\:\frac{1}{{q}_{e}}\:t$$

Where q_e_ and q_t_ (mg/g) are the amount of CV dye adsorbed at equilibrium and time *t*, respectively, K_1_ is the rate constant of PFO and K_2_ (g/mg·min) is the rate constant of PSO.

**Fig. 17:**
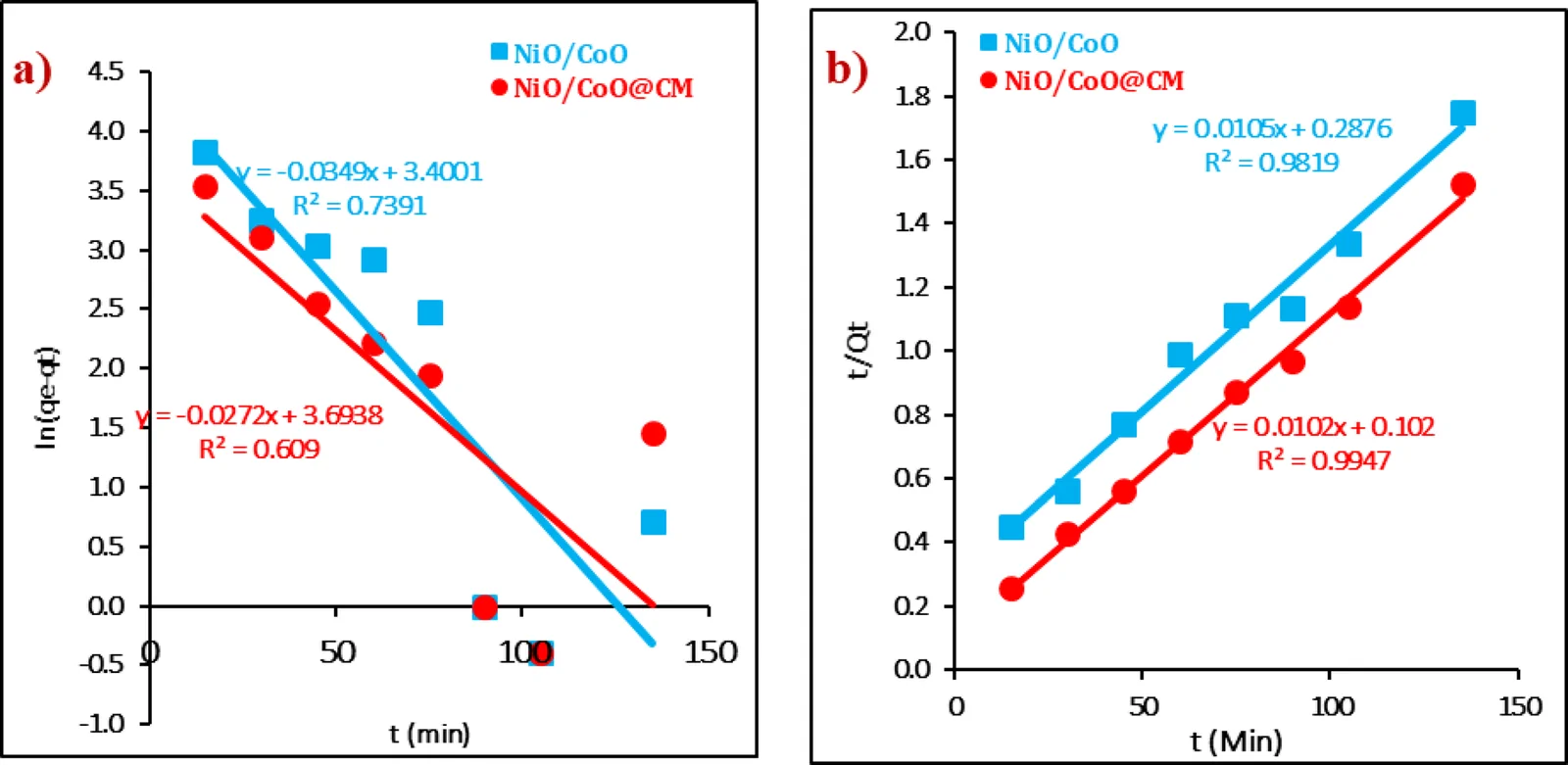
Kinetic models for CV dye adsorption dye onto NiO/CoO and NiO/CoO@CM surfaces **a**) pseudo-first order and **b**) pseudo-second order models.

The kinetic parameters for both pseudo first order (PFO) and pseudo second order (PSO) models are presented in Table (4) and illustrated in Figure (17 a&b). The PSO models exhibited a significantly better fit to the experimental data with high R^2^ values (0.982 and 0.995 for NiO/CoO and NiO/CoO@CM, respectively) compared to relatively low R^2^ values for PFO (0.74 and 0.61 for both catalysts). These results strongly revealed that the adsorption process follows chemisorption mechanism. Furthermore, the equilibrium adsorption capacities (q_e_) calculated from PSO (79.3 and 92.8 mg/g for NiO/CoO and NiO/CoO@CM, respectively) closely matched the experimentally measured values, whereas the PFO derived q_e_ values (29.96 mg/g and 40.18 mg/g, respectively) significantly underestimate the actual adsorption capacity. The PSO rate constant (K_2_) for NiO/CoO@CM (1.02 × 10⁻³ g/mg·min) was more three times higher than that for NiO/CoO (3.83 × 10⁻⁴ g/mg·min), revealing a faster adsorption rate and more efficient catalytic activity of the biogenic NiO/CoO@CM.

**Table 4:** Kinetic parameters of pseudo-first order and pseudo- second order models of CV degradation using NiO/CoO and NiO/CoO@CM catalysts.

Kientic Model	Psuedo first order reaction			Psuedo second order reaction		
**Catalyst**	**q**_**e**_ **(mg/g)**	**K**_**1**_ (min^− 1^)	**R** ^**2**^	**q**_**e**_ **(mg/g)**	K_2_ (g/mg min)	**R** ^**2**^
NiO/CoO	29.96	0.080	0.74	95.24	3.83E-04	0.982
NiO/CoO@CM	40.18	0.063	0.61	98.04	1.02E-03	0.995

## Conclusion

This study conducts a sustainable, and eco-friendly route for the synthesis of biogenic NiO/CoO nanocomposites using cyanobacterial extract, offering several benefits over conventional chemical synthesis methods. The synergistic integration of the two metal oxides with bioactive compounds from green extract, including amides, polysaccharides, and nitriles, established a novel technique for effective wastewater treatment. The biogenically synthesized catalyst exhibited superior physicochemical properties relative to chemically synthesized catalyst, including enhanced specific surface area (38.97 m^2^/g), a narrower band gap (E_g_ = 2.9 eV) and a smaller average crystallite size (27.05 nm). Under the optimized operating conditions (pH 9, contact time of 90 min, and catalyst dosage of 0.1 g/50 mL), the biogenic photocatalyst achieved a significantly higher photodegradation efficiency of 96.5% for crystal violet dye, whereas the chemically synthesized NiO/CoO achieved only 79.2% under the same conditions. Adsorption study showed that adsorption of CV on the catalyst surface is favorable fit with the Freundlich isotherm (R^2^ > 0.98; K_f_ = 39.36 mg/g) and the pseudo-second-order kinetic model (R^2^ > 0.98; q_e_ = 95.24 to 98.04 mg/g) indicating heterogeneous chemisorption during adsorption process. The enhanced photocatalytic performance of NiO/CoO@CM attributed to its efficient separation of photogenerated charge carries, and enhanced production of reactive oxygen species. Also, the biogenic catalyst showed high operational reusability (75.2% of its initial photocatalytic activity after five successive cycle associated with 87.9% TOC removal efficiency. These results ultimately establish biogenic NiO/CoO@CM nanocomposites as an affordable, high-performing, and naturally occurring substitute for traditional catalysts in the removal of organic contaminants from aquatic systems. Future investigations should focus on expanding the photocatalytic application to the degradation various organic pollutants, particularly pharmaceuticals, pesticides and phenolic compounds, using real wastewater matrices to assess scalability and practical applicability.

## Data Availability

All data sets produced and analyzed during this current study are included within the article. These data are available from the corresponding author upon request.
